# Compatibility of Evolutionary Responses to Constituent Antibiotics Drive Resistance Evolution to Drug Pairs

**DOI:** 10.1093/molbev/msab006

**Published:** 2021-02-22

**Authors:** Leonie Johanna Jahn, Daniel Simon, Mia Jensen, Charles Bradshaw, Mostafa Mostafa Hashim Ellabaan, Morten Otto Alexander Sommer

**Affiliations:** Novo Nordisk Foundation Center for Biosustainability, Technical University of Denmark, Kongens Lyngby, Denmark

**Keywords:** adaptive evolution, antimicrobial resistance, combination therapy

## Abstract

Antibiotic combinations are considered a relevant strategy to tackle the global antibiotic resistance crisis since they are believed to increase treatment efficacy and reduce resistance evolution (WHO treatment guidelines for drug-resistant tuberculosis: 2016 update.). However, studies of the evolution of bacterial resistance to combination therapy have focused on a limited number of drugs and have provided contradictory results (Lipsitch, Levin BR. 1997; Hegreness et al. 2008; Munck et al. 2014). To address this gap in our understanding, we performed a large-scale laboratory evolution experiment, adapting eight replicate lineages of *Escherichia coli* to a diverse set of 22 different antibiotics and 33 antibiotic pairs. We found that combination therapy significantly limits the evolution of de novode novo resistance in *E. coli*, yet different drug combinations vary substantially in their propensity to select for resistance. In contrast to current theories, the phenotypic features of drug pairs are weak predictors of resistance evolution. Instead, the resistance evolution is driven by the relationship between the evolutionary trajectories that lead to resistance to a drug combination and those that lead to resistance to the component drugs. Drug combinations requiring a novel genetic response from target bacteria compared with the individual component drugs significantly reduce resistance evolution. These data support combination therapy as a treatment option to decelerate resistance evolution and provide a novel framework for selecting optimized drug combinations based on bacterial evolutionary responses.

## Introduction

The prevalence of antibiotic resistance has become a global health concern, limiting the efficacy of standard treatments for acute and chronic bacterial infections (Ventola 2015). As the development of novel antibiotics is expensive in terms of time and resources (Luepke et al. 2017), it is important to use currently available drugs in the best possible way to decelerate antibiotic resistance evolution and to maximize positive treatment outcomes. Empiric combination therapy is believed to improve treatment outcomes via increased potency and reduced evolution of drug resistance (Blomberg et al. 2001; Bantar et al. 2004). However, the clinical benefit of combination therapy remains controversial (Leibovici et al. 1997; Bantar et al. 2004; Bliziotis et al. 2005; Paul 2014; Skorup et al. 2014; Tepekule et al. 2017; Lipcsey et al. 2018). The disparate results might be explained by an incomplete understanding of the factors that drive the evolution of resistance to combination therapy.

Drug combinations have been mainly studied in regards to phenotypic characteristics, such as drug interaction (Hegreness et al. 2008; Torella et al. 2010; Munck et al. 2014; Baym et al. 2016; Barbosa et al. 2018) or collateral drug responses (Munck et al. 2014; de Evgrafov et al. 2015). Drug interactions describe the combined effect of multiple drugs relative to the sum of their individual effects (additive, synergistic, and antagonistic) (Wong 2017). Collateral drug responses occur when a bacterium that evolved resistance to a drug displays higher susceptibility (collateral sensitivity) or increased resistance (collateral resistance) to other agents (Szybalski and Bryson 1952; Beutner et al. 1963). These different phenotypic characteristics have been correlated with resistance evolution in multiple studies with contradictory results ranging from accelerated to decelerated evolution (Hegreness et al. 2008; Munck et al. 2014; Barbosa et al. 2018).

In addition, the genetics underlying the resistance evolution towards drug combinations have only been studied for a very limited number of drug pairs (Munck et al. 2014; de Evgrafov et al. 2015; Suzuki et al. 2015). Two small-scale studies identified that mutations linked to collateral sensitivity were less prominent in the combination of collateral sensitive drugs (Munck et al. 2014; Suzuki et al. 2015), while another study found that the types of mutations are different in drug pair evolved lineages compared with single drug evolved ones (Laehnemann et al. 2014). Yet, the genetic trajectories towards drug combinations have not been characterized systematically under controlled conditions along with their potential to predict resistance evolution.

In order to address this lack of knowledge, we conducted a systematic high-throughput adaptive laboratory evolution (ALE) experiment for *Escherichia coli*, an important model organism and human pathogen (Bodilsen et al. 2018). The large number of replicate lineages and the broad range of drugs tested combined with a systematic assessment of the evolvability allowed us to analyze the phenotypic and genotypic evolutionary responses to single and combinatorial drug exposure. Based on this comprehensive dataset we identified for the first time distinct patterns in the genetic responses towards drug combinations. Moreover, these genetic trajectories are reliable predictors for the evolvability of antibiotic resistance.

## Results

### Resistance Evolution towards a Diverse Set of Antibiotic Combinations

**Fig. 1:**
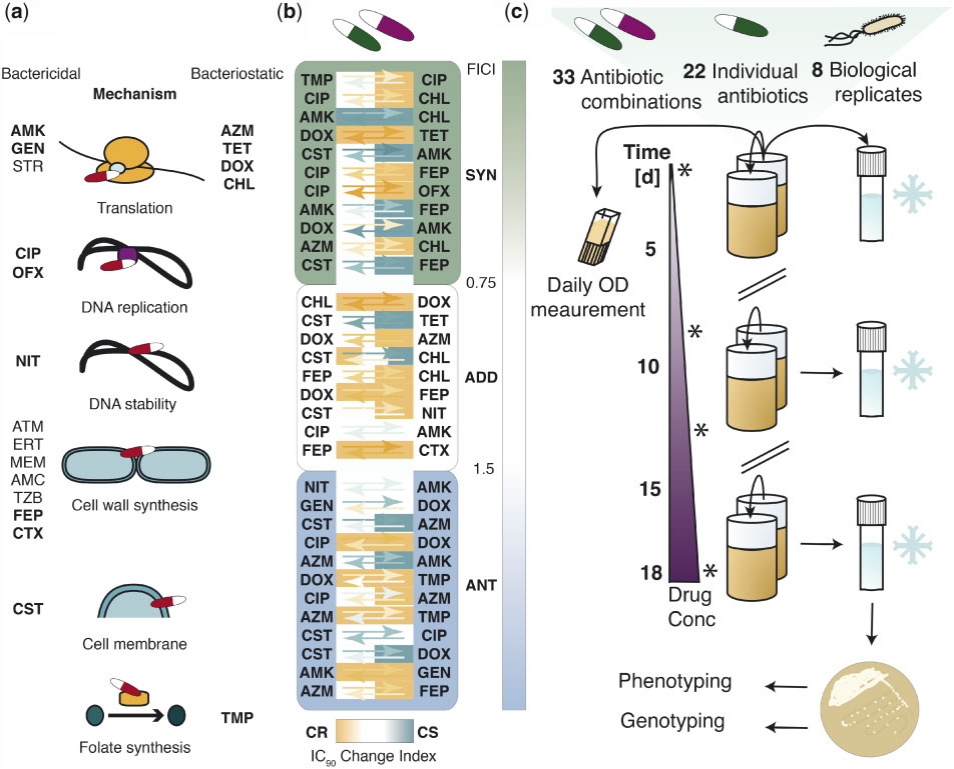
Drug properties and experimental setup. **a** Characteristics of the drugs chosen for adaptive laboratory evolution. The antibiotics were either bactericidal or bacteriostatic and covered multiple drug classes and six different processes in the cell. Drugs chosen for the evolution in drug pairs are depicted in bold. **B** The drug pairs, shown in ascending order of the fractional inhibitory concentration index (FICI), exhibit various phenotypic interactions: synergy (SYN, FICI < 0.75, green), additivity (ADD, FICI = 0.75-1.5, white) and antagonism (ANT, FICI > 1.5, blue); collateral resistance (CR, orange), a neutral collateral response (N, white) and collateral sensitivity (CS, turquoise). The arrows show the fold increase (orange, CR > 2 ∗ median ancestral wild type (WT) IC_90_) or decrease (turquoise, CS < 0.5 ∗ median WT IC_90_) in resistance compared to the WT. The direction of the arrows indicates the direction of the collateral drug response: e.g. lineages evolved to Trimethoprim display mild collateral resistance to Ciprofloxacin, while lineages evolved to Ciprofloxacin show mild collateral sensitivity to Trimethoprim. The space around the arrows is colored based on the classification of the drug pairs as CR, CS or neutral according to the collateral IC_90_ change index of each isolated biological replicate. Definitions of antibiotic abbreviations can be found in Table S2. Definitions of the different categories (SYN, ADD, ANT, CS, CR) as well as definitions of the FICI, IC_90_ and collateral IC_90_ change index can be found in materials and methods. The figure lists 32 antibiotic pairs due to the exclusion of the replicate lineages evolved to Sulfamethoxazole-Trimethoprim, as Sulfamethoxazole appeared unstable upon freezing, resulting in unreliable resistance determination. **c** Adaptive laboratory evolution of antibiotic resistance. Genetically barcoded *E. coli* lineages were evolved in eight biological replicate lineages with 22 different antibiotics and 33 different antibiotic combinations. The replicate lineages were grown in 96-deep-well-plates in 1 ml of LB containing antibiotic. Every 22 h, the cells were transferred to a new plate in a 20-fold dilution. In addition, the optical density was measured immediately before each transfer, and an aliquot of the population was saved as a glycerol stock. The evolution of resistance in each replicate lineage was monitored by measuring the IC90 at day 0, 8, 13 and 18, as indicated with stars. The evolution was started at subinhibitory drug concentrations (25 % of the WT IC90), and the WT IC90 was reached on the 7th day of the experiment. The evolution experiment ended after 18 days, when the WT IC90 was exceeded by more than 10-fold. Isolated colonies were obtained from frozen endpoints and subsequently used for whole-genome sequencing and susceptibility testing to multiple antibiotics.

To identify the underlying features that drive the evolution of resistance to combination therapy, we adapted genetically barcoded replicate lineages (Jahn et al. 2018) of the well-studied model organism *E. coli* K12 MG1655 to a diverse set of 22 different antibiotics and 33 different antibiotic pairs (supplementary tables S1 and S2, Supplementary Material online). These drugs, including both bactericidal (68.18%) and bacteriostatic (31.81%) drugs, covered 11 different drug classes and targeted 6 different bacterial processes (supplementary table S2, Supplementary Material online, fig. 1*a*). Moreover, we assessed the phenotypic features of the drug pairs. First, we classified the drug combinations based on the drug interaction and found that they covered all three possible drug interactions: synergistic (34.4%), additive (28.1%), and antagonistic (37.5%) (fig. 1*b*). The classification was done by measuring the drug concentration that resulted in a 90% growth-reduction (IC_90_) of the wild type (WT) compared with WT growth in media only for all single antibiotics and for the antibiotics in combination. Based on these values the fractional inhibitory concentration index was calculated (FICI) (Tyers and Wright 2019). While different methods to calculate drug interactions are used that impact the classification of drug interactions, we decided to use a Loewe-additivity model based on the IC_90_ (which is similar to the minimal inhibitory concentration [MIC]) as this is commonly reported in scientific studies and allows best possible comparison of our study with the existing literature (Munck et al. 2014; Gonzales et al. 2015; Stokes et al. 2017; Minato et al. 2018; Tyers and Wright 2019). Further, we defined cut-offs for the FICI (Materials and Methods) to distinguish between the drug interactions: antagonistic (ANT, FICI > 1.5), additive (ADD, FICI = 0.75–1.5), and synergistic (SYN, FICI < 0.75).

Resistance to these drugs and drug combinations was achieved via ALE (Jahn et al. 2017). Even though adaptive evolution experiments simplify the growth conditions in human hosts, they can capture clinically relevant features of resistance evolution (Imamovic et al. 2018). In addition, adaptive evolution reduces the complexity of resistance evolution in clinical settings and allows studying specific parameters systematically under controlled conditions (Jansen et al. 2013). We performed the evolution experiment in a stepwise manner (Jahn et al. 2017), in eight biological replicate lineages giving a total of 460 lineages (including 20 LB-only controls) (fig. 1*c*, methods, antibiotic concentrations in supplementary table S3, Supplementary Material online). A single isolated colony was obtained for each revived endpoint lineage for subsequent genotypic and phenotypic characterizations (supplementary table S4, Supplementary Material online). After the adaptive evolution experiment, we measured the IC_90_ of all isolates. In order to check whether the isolates were representative for the lineage they were obtained from, we calculated the difference between the IC_90_ of the lineages and the IC_90_ of the isolates derived from the respective lineages and normalized it by the lineage IC_90_, similar to the calculation of the Coefficient of variance (CV). The median of these indices was 0.66 indicating an acceptable agreement between isolates and lineage IC_90_s. However, certain antibiotics like beta-lactams and many drug combinations had higher or lower lineage resistance compared with the isolates (supplementary fig. S1, Supplementary Material online). This might be the result of different aspects, such as population dynamics (Lee et al. 2010), inoculum effect (Brook 1989), tolerance (Levin-Reisman et al. 2017), and selection bias of the isolates due to freezing sensitivity (Barbosa and Levy 2000).

We also measured the IC_90_ of all isolates adapted to single-drugs towards all single drugs used. The resulting data allowed us to assess collateral drug responses (supplementary fig. S2, Supplementary Material online). Therefore, the drug pairs could be grouped based on the collateral IC_90_ change index into one of three categories: collateral resistant (CR, collateral IC_90_ change index > 2), collateral sensitive (CS, collateral IC_90_ change index < 0.5), or neutral (N, collateral IC_90_ change index 0.5–2). The collateral IC_90_ change index provides the average change in fold resistance relative to the WT between two isolates adapted to either drug A or B to the respective other drug (Munck et al. 2014). We found that the drug pairs displayed all possible collateral responses between the individual drugs constituting the pairs (fig. 1*b*).

### Assessment of Evolutionary Responses to Combination Therapy

Before we analyzed the isolates, we also observed the behavior of the entire populations during the adaptive evolution experiment. A majority (68.4%) of the lineages adapted to monodrug exposure exhibited stable growth throughout the evolution experiments (chi-square test of independence, *X^2^* = 84.742, *P *=* *2.2*e*-16, df = 1, *n*(Mono) = 152, *n*(Combination) = 256). In contrast, most (59.4%) of the lineages exposed to drug combinations exhibited declining OD values over time (chi-square test of independence, *X^2^* = 37.028, *P *=* *1.164*e*-09, df = 1, *n*(Mono) = 152, *n*(Combination) = 256) (supplementary fig. S3*a*–*c*, Supplementary Material online). Declining OD values might indicate that the populations did not evolve resistance at a sufficient pace to ensure survival. Further, we measured the resistance level of the lineages at different time points during the experiment. We calculated the CV of the endpoint IC_90_ levels of the parallel-evolved lineages and found a significant (Mann–Whitney *U*-test, *P *=* *0.003076, *U* = 2552, two-sided, confidence level = 0.95) difference between the variance of single-drug (CV = 0.388417) and drug pair (CV = 0.6410415) evolved lineages. Usually, a higher degree of phenotypic convergent evolution is associated with a higher selection pressure and constrained evolution (MacPherson and Nuismer 2017), yet parallel evolution is also highly depended on population size (Bailey et al. 2015). As mentioned before drug pair evolved lineages had often decreasing population sizes, which might account for the higher phenotypic variability.

Looking at the IC_90_ data of the lineages during the evolution experiment, we found that after completion of the evolution a majority (67.8%) of the drug-pair-evolved replicate lineages, but a minority (23.5%) of the single-drug-evolved lineages, only gained resistance levels below the antibiotic concentration they were exposed to during the ALE (chi-square test of independence, *X^2^* = 73.117, *P *=* *2.2*e*-16, df = 1, *n*(Mono) = 152, *n*(Combination) = 256) (supplementary fig. S3*d*–*f*, Supplementary Material online). This observation suggests a limited capacity of drug-pair-exposed lineages to evolve resistance.

### Combination Therapy Reduces Resistance Evolution

To further examine resistance evolution we assessed the phenotypes of the isolated colonies from the end point of the evolution experiment. We found that isolates evolved to about half of the drug pairs (15) displayed resistance to the drug pair and the individual drugs constituting the pair. For the other drug combinations we observed resistance to the drug pair and only one of the individual drugs (eight drug pairs), only to one of the individual drugs (five drug pairs) or no resistance at all (four drug pairs).

This observation highlights variable abilities to evolve resistance and different dynamics of the drug pairs to select for adaptations.

**Fig. 2.**
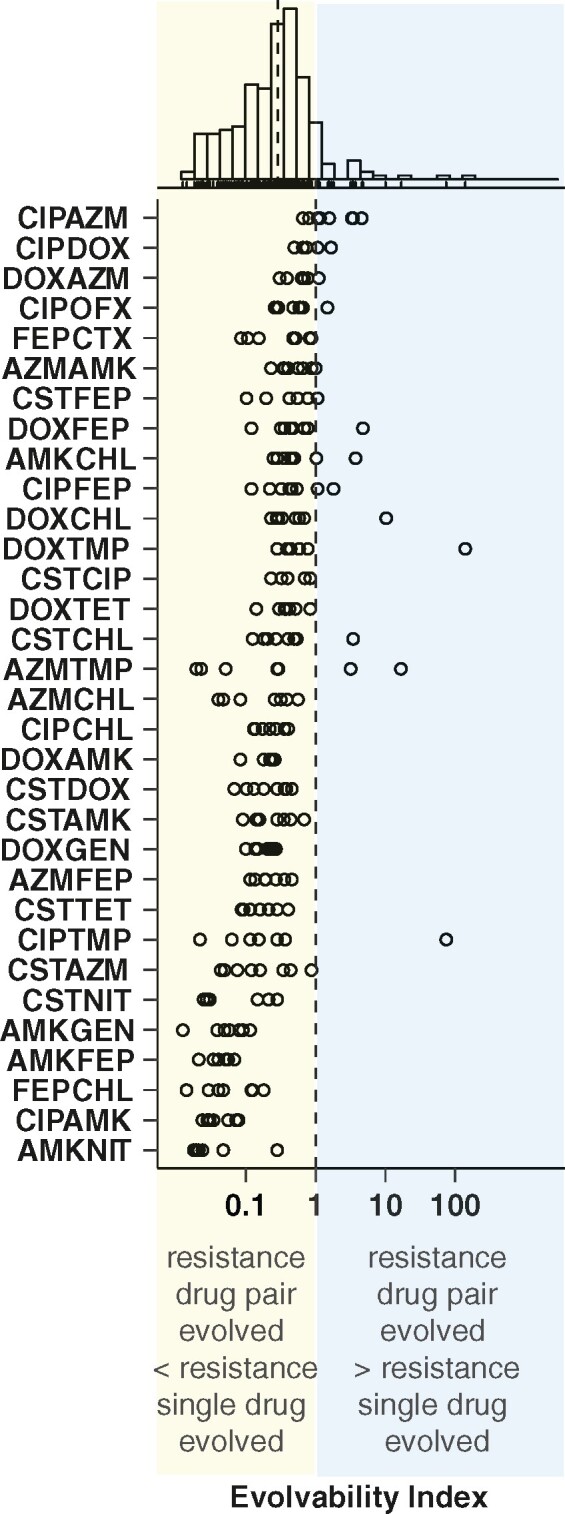
Antibiotic combinations limit resistance evolution. Distribution of the evolvability index of the different isolates from replicate lineages each represented with a dot for each drug pair. Drug pairs are ordered by median evolvability index. The histogram displays a unimodal right skewed distribution of the isolates over the evolvability index. The dotted line in the histogram indicates the median. Explanations for outliers are discussed in Table S5.

To shed light on the factors that impact resistance evolution, we calculated the evolvability index for all drug pair-evolved lineages (Munck et al. 2014). The evolvability index describes the final phenotypic adaptation level relative to single-drug-evolved isolates (Munck et al. 2014). All the isolates except those evolved to a combination of ciprofloxacin and azithromycin had a median evolvability index <1, indicating that the drug-pair-evolved isolates became less resistant to the two individual drugs than the isolates evolved to these drugs alone (fig. 2). In fact, for a majority of the drug pairs (87.5%), very limited resistance evolution was observed (evolvability index < 0.5).

These findings highlight that drug pairs in general reduce the adaptive potential of de novode novo antibiotic resistance evolution in *E. coli*. Nevertheless, *E. coli* evolved resistance to specific drug combinations to a markedly different degree.

### Phenotypic Features Impact Evolvability Only Marginally

**Fig. 3:**
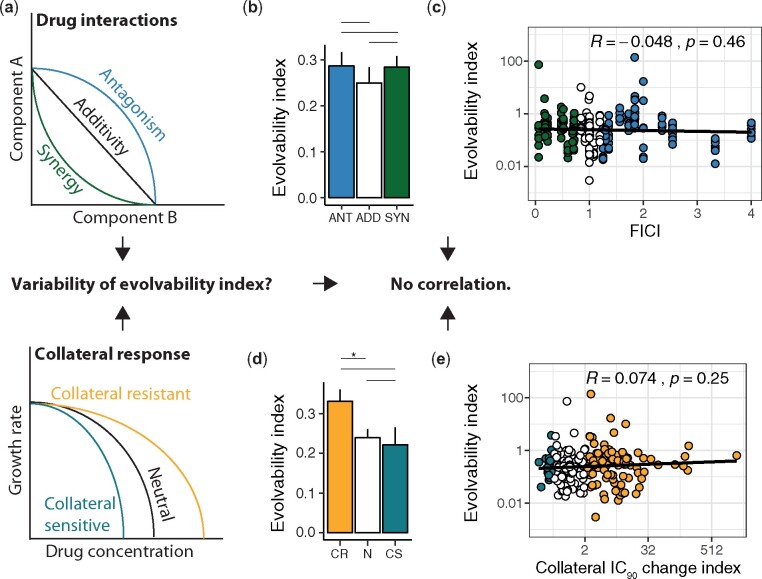
Phenotypic features of drug combinations are weak predictors of the evolvability. ANT = antagonistic drug pairs; ADD = additive drug pairs; SYN = synergistic drug pairs. CR = collateral resistance; N = neutral; CS = collateral sensitivity. **a** Schematic overview of the different types of drug interaction and collateral drug responses. It has been hypnotized previously that they might impact the evolvability of drug combinations. **b** The median evolvability indices (±MAD) for drug pairs grouped by the FICI (Mann-Whitney U-test, median(ANT) = 0.29, n(ANT) = 83, median(ADD) = 0.23, n(ADD) = 71, median(SYN) = 0.28, n(SYN) = 83, ANT-ADD: *p* = 0.3251, U = 3218.5, ANT- SYN: *p* = 0.8806, U = 3491.5, ADD-SYN: *p* = 0.3873, U = 3185.5, Bonferroni corrected, two-sided, confidence level = 0.95). **c** Scatterplot of the evolvability index versus FICI (Pearson correlation coefficient R = -0.048, *p* = 0.46). **d** Median evolvability indices (±MAD) for drug pairs grouped by the collateral IC_90_ change index (Mann-Whitney U- test, median(CR) = 0.36, n(CR) = 108, median(N) = 0.23, n(N) = 110, median(CS) = 0.22, n(CS) = 19, CR-N: *p* = 0.01654, U = 7056.5, CR-CS: *p* = 0.245, U = 1198.5, N-CS: *p* = 0.7397, U = 1095.5, Bonferroni corrected, two-sided, confidence level = 0.95). **e** Scatterplot of the evolvability index versus the collateral IC_90_ change index (Pearson correlation coefficient R = 0.074, *p* = 0.25).

Prior studies have suggested that drug features like synergistic or antagonistic drug interactions or collateral drug responses play an important role in explaining the difference in resistance evolution toward drug combinations (Hegreness et al. 2008; Munck et al. 2014; Suzuki et al. 2015; Barbosa et al. 2018) (fig. 3*a*). We grouped the drug pairs into antagonistic, additive and synergistic based on the FICI. However, we observed only a minor contribution of synergistic or antagonistic drug interactions on the evolvability of resistance (fig. 3*b*). Further, we did not find a correlation between the FICI and the evolvability index (fig. 3*c*). Next, we assessed the effect of collateral responses on evolution of resistance to drug combinations by grouping the drug pairs based on the collateral IC_90_ change index. Again, we observed only a limited effect of collateral responses on the evolvability index (fig. 3*d*) and no significant correlation between the collateral IC_90_ change index and the evolvability index (fig. 3*e*).

### Genetic Responses to Drug Pairs Follow Distinct Patterns

To determine the genetic basis of resistance and to assess if the genotypes could explain the varying levels of evolvability among the different drug pairs, we performed whole-genome sequencing on 313 of the phenotypically characterized isolates that exhibited phenotypic resistance (IC_90_ > 2-fold WT IC_90_) after an initial screening (supplementary table S6, Supplementary Material online). In total, we found 1,062 single nucleotide variants, 1,052 gene duplications, and 368 insertions or deletions (supplementary table S7, Supplementary Material online). Six isolates displayed a hypermutator phenotype with between 21 and 383 mutations. All hypermutators had a mutation in either *mutS, mutt*, or *mutD* (*dnaQ*) (supplementary table S7, Supplementary Material online), which induce the hypermutator phenotype (Jolivet-Gougeon et al. 2011). On average, we detected ∼5 mutations per isolate. The number of mutations per isolate was roughly the same between isolates adapted to a single or to multiple antibiotics (supplementary fig. S4, Supplementary Material online). However, the types of mutations differed. While single nucleotide polymorphisms (SNP) were the dominant response under single drug exposure, gene duplications were most prevalent in drug pair evolved isolates, as reported before (Laehnemann et al. 2014). The gene that was mutated the most (101 times + 15 times in the promoter region) was *marR* (supplementary table S7, Supplementary Material online), the regulator of the multiple antibiotic resistance locus (Cohen et al. 1993), a gene in which mutations can induce a multidrug resistance phenotype (Woodford and Ellington 2007). Antibiotic resistance is facilitated through MarR by the transcriptional regulation of at least 80 chromosomal genes (Pomposiello et al. 2001; Barbosa and Levy 2000; Alekshun and Levy 1997), involving primarily stress response (Alekshun and Levy 1997) and multidrug efflux (Keeney et al. 2008). Multiple other mutations are also known to be linked to a multidrug resistance phenotype and often involve AcrB-mediated efflux of the antibiotic (Okusu et al. 1996) and have been identified in this study (supplementary table S7, Supplementary Material online). The multidrug resistance induced through these mutations can be illustrated by clustering the antibiotic evolved isolates based on their genetic similarity. We calculated the genetic similarity of all single drug evolved isolates based on the Jaccard’s Distance and found that isolates evolved to different drugs like tetracyclines, chloramphenicol, beta-lactam and macrolide antibiotics clustered together (supplementary fig. S5, Supplementary Material online). This finding highlights that resistance mechanisms evolve that are not necessarily specific to the mechanism of action of the antibiotic. In addition, the genetic similarity can also explain collateral resistance as genetic similarity and collateral resistance are positively correlated (supplementary fig. S6, Supplementary Material online).

**Fig. 4.**
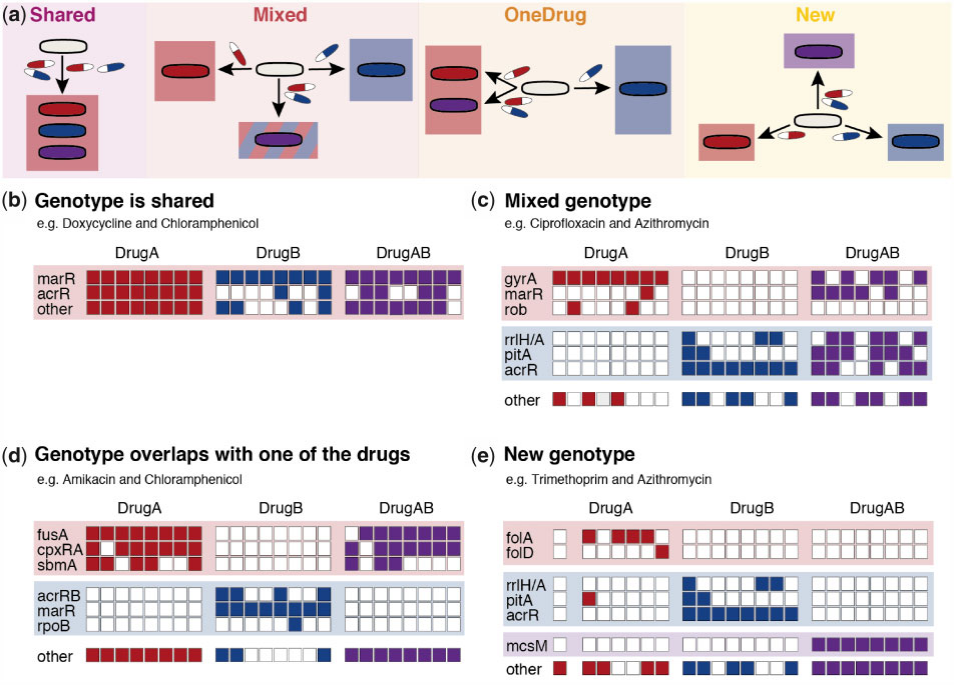
Drug pairs can be grouped in four distinct categories based on their genotypic response in relation to the genotype of isolates adapted to the constituent drugs. **a** Schematic overview of the possible genetic responses of drug-pair-evolved isolates compared to those evolved to the component drugs. **b** – **e** Examples of the genotypes of the eight replicates of single-drug-evolved and drug-pair-evolved isolates for each genetic group.

We grouped drug-pair-evolved isolates into four distinct genetic responses relative to their genetic response towards their constituent drugs: 1) mutations conferring resistance to both constituent drugs are the same and are selected by the drug combination (Shared genotype); 2) mutations conferring resistance to both constituent drugs are different, yet are both selected by the drug combination (Mixed genotype); 3) mutations conferring resistance to both constituent drugs are different, yet only mutations for one of the constituent drugs are selected by the drug combination (One Drug genotype); or 4) mutations selected by the drug combination are different from those selected by each of the constituent drugs (New genotype) (fig. 4*a*). To classify the drug pairs into these distinct categories, we performed an analysis of similarities (ANOSIM) based on the mutations of each sequenced isolate (supplementary table S7, Supplementary Material online). ANOSIM is a nonparametric statistical test that is widely used in ecology to identify differences among ecological niches based on ranked dissimilarity matrices (Bueno et al. 2018). Here, we used mutated genes as features to identify differences between various adaptation conditions instead of characteristics of an ecological niche.

The Shared group contained three drug pairs for which no significant genotypic differences (*R* < 0.2 and/or *P *>* *0.005, supplementary table S8, Supplementary Material online) were observed between single-drug-evolved isolates or between single-drug-evolved isolates and drug-pair-evolved isolates (fig. 4*b*). For example, key mutations found in doxycycline-adapted isolates were also dominant in chloramphenicol-evolved isolates as well as isolates exposed to both drugs simultaneously. All drug pairs belonging to the Shared group exhibited collateral resistance to each other (supplementary fig. S7, Supplementary Material online), as the genetic alterations provide resistance to both individual drugs as well as to the drug pair.

The Mixed group contained two drug pairs, where a significant difference (*R* > 0.2 and *P *<* *0.005, supplementary table S8, Supplementary Material online) between the genotypes of isolates evolved to individual drugs was observed, but no significant difference was observed between the genotypes of drug-pair-evolved isolates and those of isolates evolved to individual drugs (fig. 4*c*). For example, while the genotypes of isolates evolved to either ciprofloxacin or azithromycin were completely different, the drug-pair-evolved isolates exhibited key mutations that were also found in the isolates exposed to the individual drugs (fig. 4*c*). This drug combination was also the only one that had a median evolvability index >1, indicating that compatible genetic pathways are unlikely to reduce the evolvability. The two drug pairs with a Mixed genotype exhibited either neutral or collateral resistance to each other, further highlighting that these drug pairs have compatible genetic responses (supplementary fig. S7, Supplementary Material online).

The One Drug group was composed of drug pairs where the genotype exclusively resembled that of isolates evolved to one of the individual drugs and contained 14 drug pairs (fig. 4*d*). For example, mutations selected against amikacin were also present in the isolates exposed to amikacin and chloramphenicol, while none of the mutations found in chloramphenicol-adapted isolates were selected in the drug-pair-evolved isolates. Five drug pairs in the One Drug group reached only final exposure levels around the IC_90_ of the individual drugs, mainly due to synergism (supplementary fig. S7, Supplementary Material online). This finding indicates that adaptation to highly synergistic drug pairs might be achieved by selection of mutations against one of the constituent drugs thereby possibly shifting the drug interaction profile. This would suggest that synergistic drug combinations could readily loose efficiency if used at insufficient doses, as previously suggested (Lipsitch and Levin 1997; Pena-Miller et al. 2013). Other factors influencing the selection of mutations towards one of the drugs might be differences in the steepness of the dose response curves (Chevereau and Bollenbach 2015) and accordingly different levels of selection pressure applied by the two drugs (supplementary fig. S8, Supplementary Material online) as well as differences in the mutation selection window and the ability to select for mutations at subinhibitory concentrations (supplementary fig. S9, Supplementary Material online).

Another five of the drug combinations in the OneDrug group were resistant to both individual drugs and drug pairs. Drugs in these pairs were substrates of the AcrB efflux pump (Yu et al. 2003). While isolates adapted to one of the constituent drugs alone, such as ciprofloxacin, develop resistance primarily via other resistance modes, such as mutations in *gyrA*, isolates adapted to the other drug, such as doxycycline, select for efflux-enhancing mutations. In combination, the efflux mutations are dominant, as these mutations confer resistance to both drugs simultaneously and are therefore likely to be selected. Consequently, the resulting genotype resembles the genotype of the efflux-mutation-selecting single-drug-evolved isolates, even though a shared resistance mechanism is selected. Of the remaining drug combinations in the OneDrug group, two displayed collateral sensitivity, which might have suppressed resistance evolution to one of the drugs.

The New group included drug pairs for which the ANOSIM gave significant (*R* > 0.2, *P *<* *0.005, supplementary table S8, Supplementary Material online) differences in genotypes between individual drugs and drug pairs (fig. 4*e*). For example, azithromycin- and trimethoprim-adapted isolates shared almost no mutations, while the isolates evolved to the combination of azithromycin and trimethoprim selected none of these mutations but repeatedly accumulated mutations in the mechanosensitive channel encoding gene *mscM.* Drug pairs with collateral sensitivity were found only in the OneDrug and New groups (supplementary fig. S7, Supplementary Material online), highlighting that those incompatible genetic trajectories to the individual drugs cannot be co-selected in drug combinations.

### Drug Pairs Requiring Novel Genetic Responses Exhibit the Lowest Evolvability Index

**Fig. 5:**
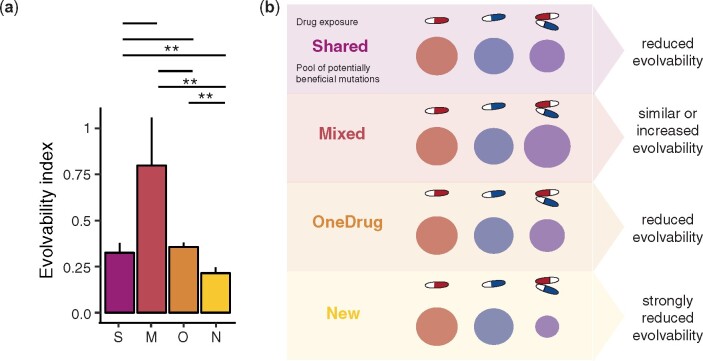
Drug pairs requiring a novel genetic response compared to the constituent drugs have a significantly lower resistance evolvability. S = Shared genotype; M = Mixed genotype; O = OneDrug genotype; N = New genotype. **a** The median evolvability index (±MAD) of drug combinations grouped by genetic response patterns (Mann-Whitney U- test, median(Shared) = 0.32, n(Shared) = 24, median(Mixed) = 0.8, n(Mixed) = 15, median(OneDrug) = 0.36, n(OneDrug) = 99, median(New) = 0.21, n(New) = 39, Shared- Mixed: *p* = 0.1308, U = 233, Shared-OneDrug: *p* = 0.5815, U = 1146, Shared-New: **p**= 0.008947, U = 291, Mixed-OneDrug: *p* = 0.0697, U = 997.5, Mixed-New: *p* = 0.009358, U = 438, OneDrug-New: *p* = 0.002111, U = 1376, Bonferroni corrected, two-sided, confidence level = 0.95). **b** Schematic overview of the different drug regimes (single drug A, single drug B and drug pair A and B) and the pool of potentially beneficial mutations that can confer resistance. We hypnotize that the adaptation potential of the Shared and OneDrug groups are comparable to the ones of the individual drugs, maybe slightly smaller due to few genetic constrains. The pool of beneficial mutations of the mixed grou*p* might in fact be bigger than the two pools of the single drugs as mutations against both drugs can be selected for. This might explain why the evolvability of drug pair evolved isolates in the mixed grou*p* can be higher than the evolvability of single drug evolved isolates. The New grou*p* has a much smaller pool of beneficial mutations, as the adaptations against the individual drugs are not compatible, reducing the options for adaptations and therefore the evolvability.

To assess the impact of genotypic response on phenotypic evolvability, we analyzed the evolvability of the four different genetic groups. Drug pairs in the New group generally showed a lower evolvability index and a lower evolutionary rate (fig. 5*a*). Of the five drug pairs that composed the New genotype group, three exhibited collateral sensitivity to each other in at least one direction and two were defined collateral sensitive based on the collateral IC_90_ change index. All three contained an aminoglycoside antibiotic (supplementary fig. S7, Supplementary Material online). These drug pairs also had the lowest evolvability indices within the group. However, isolates evolved to azithromycin and trimethoprim also developed a distinct new genotype, despite a lack of collateral sensitivity.

Overall, these findings highlight that drug combinations work best at decelerating resistance evolution when the resistance modes to the individual drugs are incompatible and require a novel genetic response (fig. 5*b*). There appears to be a low probability of selection of these novel responses, as evolvability in this genetic group was significantly lower than that in the other groups (fig. 5). Interestingly, collateral sensitivity might be an indicator for the genetic incompatibility as drug pairs with collateral sensitivity grouped exclusively in the New and OneDrug group (Pearson's Chi-squared test, *X^2^* = 16.381, *P *=* *0.01185, df = 6). By contrast, drug pairs that evolved resistance by selecting for mutations against both drugs, belonging either to the Mixed or Shared group and in part to the OneDrug groups, had higher evolvability indices (fig. 5*a*), demonstrating that combinations of antibiotics that have compatible genetic responses are not well suited to limit resistance evolution.

## Discussion

This study aimed to assess the potential of antibiotic combinations in reducing resistance evolution and to identify key properties of these combinations that can predict resistance evolution. We observed that de novode novo antibiotic resistance evolution is reduced in *E. coli* when two antibiotics are combined. We further assessed the ability of phenotypic parameters, such as drug interactions and collateral responses to predict the evolvability.

Previous studies reported conflicting abilities of synergistic or antagonistic drug interactions in limiting resistance evolution (Lipsitch and Levin 1997; Hegreness et al. 2008; Torella et al. 2010; Pena-Miller et al. 2013; Munck et al. 2014; Barbosa et al. 2018). In line with Munck et al. (2014), we find that drug interactions are weak predictors for resistance evolution. In addition to drug interactions we also analyzed the impact of collateral drug responses on the evolvability of resistance to drug combinations. Previous work had shown a correlation between collateral sensitivity and limited resistance evolution. However, even though drug pairs with collateral sensitivity had a lower evolvability index as neutral or collateral resistant drug pairs, the difference was not significant. This could be due to the small sample size of collateral sensitive drug pairs or the experimental design that selected for a specific resistance level.

Yet, by systematically analyzing the genetic adaptations, we observed a clear pattern relating the genetic trajectories to resistance evolution. Grouping of drug pairs based on genotypes revealed that resistance evolution to drug pairs that required a new genotypic response relative to the genetic adaptations to the constituent drugs was greatly limited. Future work in identifying further evolutionary constrained drug pairs and a framework to predict limited resistance evolution will be needed in order to identify the best drug combinations for limited resistance evolution.

In general, our data provides a comprehensive resource for the exploration of de novode novo resistance evolution in *E. coli* and of the different phenotypic and genotypic adaptations to monotherapy and combination therapy. However, the number of isolated colonies for each evolved lineage could be expanded in order to ensure that population heterogeneity and heteroresistance is captured sufficiently in the analysis and additional drug combinations and organisms would need to be characterized to elucidate whether our findings can be further generalized. In addition, the impact of drug combinations on the evolution of antibiotic tolerance should be addressed in future work. Moreover, it remains to be determined whether our findings can be translated to the clinic. Adaptive evolution is frequently used to explore the response to antibiotic exposure (Imamovic and Sommer 2013; Lázár et al. 2013; Munck et al. 2014; Jahn et al. 2017). However, factors, such as horizontal gene transfer, host–pathogen interactions, interactions between bacterial populations, side effects and pharmacodynamics of the antibiotics, as well as patient condition and disease, need to be considered when clinical experiments are conducted. Nonetheless, we expect that this framework for assessment of evolvability of drug combinations will be the base for further research on the rational design of drug combinations for efficient and resistance-limiting therapies.

## Materials and Methods

### Bacterial Strains and Growth Conditions

Chromosomally barcoded *E. coli* MG1655 K12 (Jahn et al. 2018) were grown in LB at 37 °C and 600 r.p.m. shaking. They were grown under the same conditions without shaking for the IC_90_ determination.

### ALE to Individual Drugs and Drug Combinations

*Escherichia coli* lineages were evolved each in eight biological replicate lineages to 22 different antibiotics and 33 different antibiotic pairs (supplementary table S1, Supplementary Material online) resulting in 460 individual lineages of which all *E. coli* lineages carried a unique genetic barcode (Jahn et al. 2018). Barcodes allowed to track lineages and to ensure that no cross-contamination between replicates took place. Moreover, genetically adapted lineages with barcodes can be provided as a valuable resource for additional experiments. All antibiotics used in this study, their mechanism of action, solvent and storage conditions are listed in supplementary table S2, Supplementary Material online. For the evolution towards drug combinations, the drugs were combined in a 1:1 ratio based on the WT IC_90_ values of the individual drugs (Munck et al. 2014). The WT was exposed to a dilution series of the drug mixture, the IC_90_ of the drug combination was established and used as a reference to define the antibiotic concentrations used for the adaptive evolution experiment (supplementary table S3, Supplementary Material online). The antibiotic pairs were chosen to cover the most important drug classes (beta-lactams, flourquinolones, aminoglycosides, macrolides, tetracyclines, chloramphenicol, and peptide antibiotics) in all their possible combinations, to include drug pairs of drugs from the same drug classes and some additional drug pairs so that we could cover all possible drug interactions and collateral relationships between drug pairs.

The adaptive evolution experiment was carried out in 96-deep-well plates. The plates were filled with LB by a Hamilton robot, sealed and stored at room temperature. Antibiotics were added by the robot the day before the experiment started and plates were stored at –20 °C. An overnight culture grown in LB was used to inoculate the ALE experiments. All passaging of cells was done manually with an 8-channel pipette. As a control 20 replicates were evolved to LB media alone. Each 96-well plate also harbored eight negative controls that stayed uncontaminated throughout the evolution experiment. Cells were grown for 22 h at 37 °C and 600 r.p.m. shaking, ensuring mixing of the population and aerobic growth conditions (aerobic growth conditions during the evolution can be assumed as genetic adaptations to aminoglycoside antibiotics, whose uptake depends on aerobic respiration, were identified as well as a mutational overlap with other studies that had a greater surface: volume ratio [Munck et al. 2014] or better mixing [Hegreness et al. 2008]). Thereafter, 100 μl were transferred to a 96-well plate and the optical density (OD_600_) of each lineage was measured in an ELx808 Absorbance Reader (BioTek) at a wavelength of 600 nm. In addition, 50 μl of cells, corresponding to a 20-fold dilution (Wahl et al. 2002; Jahn et al. 2017), were passaged to a new preheated 96-deep-well plate containing LB and a 25% increase in antibiotic concentration in a total volume of 1 ml/well. The starting concentration was 25% of the WT IC_90_ and the WT IC_90_ drug concentration was reached on the seventh day of the ALE (supplementary table S3, Supplementary Material online). The evolution was stopped after 18 days at a final concentration exceeding 10-fold of the WT IC_90_ (Jahn et al. 2017). All antibiotic concentrations can be found in supplementary table S3, Supplementary Material online. The IC_90_ of the lineages was measured on day 0, 8, 13, and 18 of the ALE in order to track the resistance evolution on the population level.

After each transfer an aliquot of 100 μl was mixed with glycerol to a final glycerol concentration of 12.5% and stored at –80 °C. Cells were streaked on LB agar from the frozen aliquot saved on the last day with growth (OD_600_ > 0.1). Some cells were very difficult to revive as observed before (Barbosa et al. 2017). If reviving failed, cells were inoculated into liquid LB before being streaked on LB agar. If cells still failed to revive, cells were streaked from the aliquot saved the day before the last day of growth. Despite the effort, some lineages would not revive at all. A list with all lineages, their last day of growth in the ALE and the day of the ALE they have been revived from can be found in the supplementary (supplementary table S4, Supplementary Material online). One isolated colony was picked randomly for each evolved lineage, grown in LB and frozen at –80 °C for further phenotypic and genotypic characterization. Lineages adapted to the following antibiotics: Erythromycin, Sulfamethoxazole, Fosfomycin as well as the combination of Sulfamethoxazole and Trimethoprim displayed inconsistent phenotypes or did not develop resistance due to technical reasons, such as drug stability after freezing. Therefore, these drugs were excluded from this study.

### IC_90_ Determination

100 μl of LB were inoculated with pin-replicators from frozen stocks of isolated colonies and grown overnight. About 10^5^ cells were transferred with pin-replicators into plates containing a 2-fold drug gradient ranging over ten different concentrations. Plates were grown at 37 °C for 18 h. The OD_600_ was measured for each well. The OD_600_ data were normalized and used to create dose-response curves in R as described before (Munck et al. 2014). In brief, percent inhibition was calculated by the following formula:

$$1-\;\frac{{OD}_{600\;\mathrm{drug}\;\mathrm{exposed}}-{OD}_{600\;\mathrm{blank}}}{{OD}_{600\;\mathrm{media}\;\mathrm{exposed}}-{OD}_{600\;\mathrm{blank}}}$$

The IC_90_ was defined as the lowest concentration of the drug that inhibited 90% of the growth (Munck et al. 2014; Imamovic et al. 2018). All IC_90_ values were generated at least in two technical replicates. If the WT IC_90_ differed >2-fold from the WT IC_90_ value established before the ALE started, the IC_90_ test was repeated along with the ancestor WT. No significant (Student’s *t*-test, *P *>* *0.05) differences between the susceptibility of the WT and the media adapted WT were observed. The IC_90_ values were normalized to the media adapted WT IC_90_. The heatmap presenting the collateral sensitivity and resistance of the single drug evolved lineages (supplementary fig. S2, Supplementary Material online) displays the times increase of the IC_90_ compared with the media adapted WT with a significance level of at least *P *<* *0.0001. Significance levels were obtained as described before (Imamovic et al. 2018). Briefly, by comparing the growth data OD_600_ in ten different antibiotic concentrations of all technical and biological replicates adapted to the same drug, with all media adapted technical and biological replicates exposed to the same drug and concentration. Within the natural variation of the samples 3,000 additional data points were computed to identify robust differences among samples. Times increase or decrease in growth compared with the WT was calculated in steps of 0.5 ranging from –10.5 to 10.5. Pairwise *t*-tests between drug adapted and media adapted data were performed and the highest times increase/the lowest times decrease with a significance value of at least *P *<* *0.0001 was given as output.

### Calculation of Important Variables

Based on the IC_90_ values several calculations were made, that are explained in the following:

The evolvability index is a measure of the final phenotypic adaptation level of isolated drug-pair-evolved lineages to the individual drugs relative to isolated single-drug-evolved lineages (Munck et al. 2014). The evolvability index compares resistance evolution between drug pair and single drug evolved lineages to individual antibiotics. It was calculated as described before (Munck et al. 2014). In short, the following formula was used:

$$\mathrm{evolvability}\;\mathrm{index}=\;\frac{\begin{array}{c} \left({IC}_{90}A_{AB}/{IC}_{90}A_{WT})/({IC}_{90}A_{A}/{IC}_{90}A_{WT}\right)\\ +({{IC}_{90}B_{AB}/IC}_{90}B_{WT})/({IC}_{90}B_{B}/{IC}_{90}B_{WT})\; \end{array}}{2}$$

Two replicate lineages evolved to Amikacin and Nitrofurantion, as well as two replicate lineages evolved to Ciprofloxacin and Doxycycline had evolvability indices above 1000. They displayed extremely high IC_90_ values, when tested to one of the individual drugs (Nitrofurantion/Doxycycline). As these values were way outside of a reasonable range of resistance they were treated as technical errors and therefore excluded from the entire analysis.

Drug interactions were determined for isolated colonies using a Loewe additivity model and the IC_90_ as effect level. The Loewe additivity model was chosen as it assumes additive effects of identical drugs (Munck et al. 2014). This is important as drugs with the same target and drugs from the same drug class were combined in this experiment. The FICI was calculated according to the following formula:

$$\mathrm{FICI}=\;\frac{{IC}_{90}{AB}_{WT}*\;\omega }{{IC}_{90}A_{WT}}+\frac{{IC}_{90}{AB}_{WT}*(1-\omega )}{{IC}_{90}B_{WT}}$$

*ω* is the molar fraction of *A* in the drug combination *AB*. As it was shown that additive effects are robustly detected at a cutoff between 1 and 1.25 (Meletiadis et al. 2010), we applied a low but symmetric cutoff in order to group the drug pairs into synergistic (<0.75), antagonistic (>1.5), and additive (0.75–1.5) combinations.

The collateral IC_90_ change index was calculated for isolated colonies as described before (Munck et al. 2014). In short, the following formula was used:

$${\mathrm{collateral}\;IC}_{90}c\mathrm{hange}\;\mathrm{index}=\;\frac{{IC}_{90}A_{B}/{IC}_{90}A_{WT}+{IC}_{90}B_{A}/{IC}_{90}B_{WT}\;}{2}$$

All drug pair evolved lineages were grouped into collateral sensitivity (< 0.5), collateral resistance (>2) and neutral (0.5–2) effects according to the collateral IC_90_ change index.

A table including all phenotypic information of the drug pair evolved lineages can be found in the supplement (supplementary table S9, Supplementary Material online).

### Whole-Genome Sequencing and Sequence Analysis

1 ml LB in each well of a 96-well, deep-well plate was inoculated from frozen stocks of isolated colonies and grown at 37 °C and 600 r.p.m. overnight. Cells were spun down at 2,000 r.p.m. for 3 min. LB was removed and replaced by DNA shielding buffer (Zymo Research). Samples were sent to BaseClear B.V. for genomic DNA extraction (ZYMO research), Nextera XT library preparation (Illumina) and 125 paired-end whole-genome Illumina HiSeq 2500 sequencing. The resulting fasta reads were used in the following workflow:

1. Single nucleotide variants (SNPs) and small insertions and deletions (INDELS) were called using CLC Genomics workbench as described before^40^. *Escherichia coli* reads were aligned to the *E. coli* K12 U00096 reference genome. On average, the coverage/base was at least 37-fold. For SNP calling only positions with a phred score of at least 30 at the position where the SNP occurred and at the three neighboring positions were considered. In addition, the SNP had to be detected with a frequency of at least 80%.
2. CLC Genomics workbench was further used to detect large insertions and deletions (large INDELS) in the reads using the INDEL function at default settings. The resulting INDELS were considered when they occurred with a frequency of >80% and in >5 different reads.
3. Large insertions were additionally detected by a custom made script used before^41^. The reference genomes of MG1655 as well as all open reading frames were downloaded from the NCBI nucleotide archive and used to cluster all ORFs with cd_hit (Li and Godzik 2006). The cluster cut off was 90% identity and coverage. Afterwards the sequenced reads from this study were quality filtered using the FASTX-Toolkit package with a minimum quality of 30 and blasted against the clustered ORFs with a word size of 16 and an *e*-value of 0.01. Reads, with >90% coverage mapping continuously to the genome, that mapped to two different clusters with an overlap between 30% and 70% were kept for further analysis. Reads were filtered so they did not cover clusters representing adjacent genes. Finally, large insertions were only counted when they were observed in at least 5 individual reads.
4. Gene duplications were detected using CLC workbench and a customized script in R as described before(Jahn et al. 2017). Regions > 100 bp of significantly (*P* < 0.00001) increased coverage according to a Poisson distribution were identified using CLC workbench. The identified regions were mapped to the genome and a gene that was overlapping at least 95% with a region of high coverage was counted as gene duplication.

INDELS that were detected by multiple of the parallel analyses were only counted once. Seven WT lineages adapted to the media were sequenced as a control and mutations as well as duplications found in these lineages were excluded from all lineages as they are likely mutations that have been inserted prior to the experiment or are involved in media adaptations. As no significant (Student’s *t*-test, *P *>* *0.05) phenotypic difference between the resistance level of the ancestor WT and the media adapted WT lineages were identified, those genetic changes are unlikely to cause antibiotic resistance.

### Jaccard’s Distance

The genetic data were used to create a presence absence table for each mutations and lineage. Based on this matrix the Jaccard’s distance was calculated using the jaccardSets function in R from the package bayesbio (McKenzie 2016).

### Analysis of Similarity

Based on the genetic data including SNPs, INDELS and gene duplications, a binary presence absence data matrix was created for each lineage and all genes. The matrix was summed for all replicates of the same condition and subsequently used to calculate a dissimilarity matrix with the package “vegan” in R using Euclidian distance (Oksanen et al. 2019). We performed an ANOSIM with the anosim function from the package “vegan” in R for the entire dataset in order to test whether significant differences between groups could be expected (Oksanen et al. 2019). Our dataset included significant (*P *<* *0.01) differences between lineages adapted to different drugs, wherefore we calculated pair-wise differences between different drug-adapted groups of replicates with the same methodology and 1,000 permutations. We calculated three different similarities for each drug pair: first, we compared lineages evolved to both single drugs constituting the pair, second, we compared the group of one of the single drug adapted lineages to the drug pair evolved lineages and third, we compared the other single drug evolved lineages to the drug pair evolved ones. Groups were considered to be significantly different when they had a *P*-value <0.005 and an R statistics >0.2. A R statistics of 0.2 has previously been described as measure for mild similarities between groups (Oksanen et al. 2019). The results were aggregated with the package “data.table” (Dowle and Srinivasan 2018).

### Data Availability and Code

Genomic data are available in NCBI under the accession number SUB5823083. All phenotypic data and scripts can be provided upon request. For the calculations and different analysis the following R packages have been utilized: “plyr” (Wickham 2011), “dplyr” (Wickham et al. 2018), “tidyr” (Wickham and Henry 2018), “ggplot2” (Wickham 2016), “data.table” (Dowle and Srinivasan 2018), “gdata” (Warnes et al. 2017), “SciViews” (Grosjean 2018), “drc” (Ritz et al. 2015), “scales” (Wickham 2018a), “gridExtra” (Auguie 2017), “cowplot” (Wilke 2018), “stringr” (Wickham 2018b), “ggpubr” (Kassambara 2018), “magrittr” (Bache and Wickham 2014). For figure 1, RawGraphs (Mauri et al. 2017) was used to create the figure. All figures were edited in Abode Illustrator.

## Supplementary Material

Supplementary data are available at *Molecular Biology and Evolution* online.

## Supplementary Material

msab006_Supplementary_Data
