# Supplementary material for: Compatibility of Evolutionary Responses to Constituent Antibiotics Drive Resistance Evolution to Drug Pairs
Source: Mol Biol Evol. 2021 Feb 22;38(5):2057–69. doi: 10.1093/molbev/msab006 (PMC8097295; doi:10.1093/molbev/msab006)
Supplement: msab006_Supplementary_Data [file msab006_supplementary_data.zip › SupplementaryInformation.docx]

**Supplementary Information**

**Fig. S1**

**
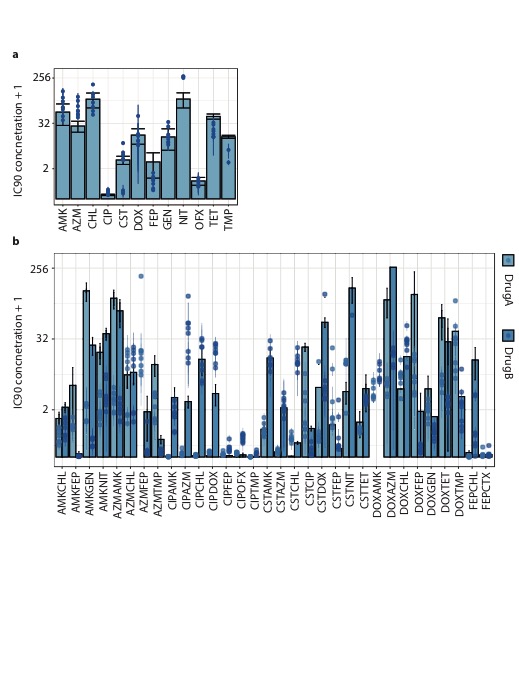
**

**Figure S1:** The median IC_90_ values of the replicate lineages measured immediately after the end of the adaptive evolution experiment are displayed in bars. Points correspond to the IC_90_ of the isolates obtained from the respective lineages. **a** Single drug evolved lineages and isolates evolved to antibiotics that were also used to formulate drug pairs. **b** Drug pair evolved lineages and isolates.

**Fig. S2**

**Figure S2: a** Collateral drug responses. Collateral interaction heatmap of all individual drug-evolved lineages tested against all individual antibiotics. Orange indicates a significant (*p* < 0.0001) fold increase in resistance compared to the wild type (WT) population, while turquoise indicates collateral sensitivity. Abbreviations of antibiotics are explained in Table S2. **b** Significant differences (ANOVA, *p* < 0.05/0.01) between the replicate isolates evolved to a single drug and tested against all individual antibiotics.

**Fig. S3**

**
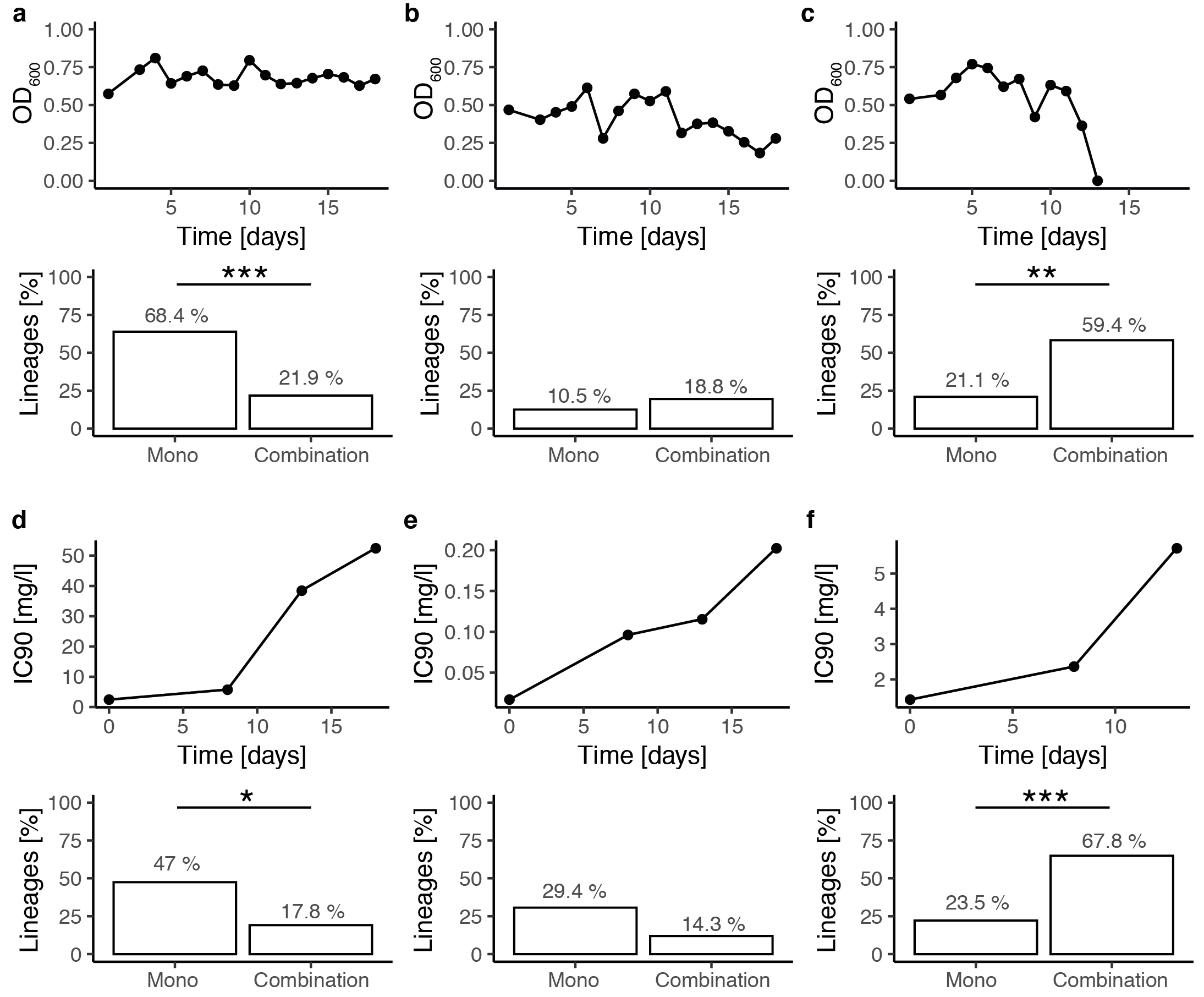
**

**Figure S3:** Distribution of optical density and population IC_90_ patterns during the adaptive evolution experiment. **a** Example of stable OD over the experiment (upper panel) (lineage 7 adapted to Ciprofloxacin) and the percentage of populations exposed to mono and combination therapy that exhibited that pattern (lower panel). **b** Example of decrease in OD after the WT IC_90_ was reached (upper panel) (lineage 3 adapted to Gentamicin) and the percentage of populations exposed to mono and combination therapy that exhibited that pattern (lower panel). **c** Example of declining OD (upper panel) (lineage 3 adapted to Amoxicillin/Clavulanic acid) and the percentage of populations exposed to mono and combination therapy that exhibited that pattern (lower panel). **d** Example of resistance adaptation at or above the antibiotic exposure level (upper panel) (lineage 8 evolved to Amikacin) and the percentage of populations exposed to mono and combination therapy that exhibited that pattern (lower panel). **e** Example of resistance adaptation at subinhibitory drug concentrations (upper panel) (lineage 3 evolved to Ciprofloxacin) and the percentage of populations exposed to mono and combination therapy that exhibited that pattern (lower panel). **f** Example of resistance adaptation below the antibiotic exposure level (upper panel) (lineage 6 evolved to Amoxicillin/Clavulanic acid) and the percentage of populations exposed to mono and combination therapy that exhibited that pattern (lower panel).

**Fig. S4**

**
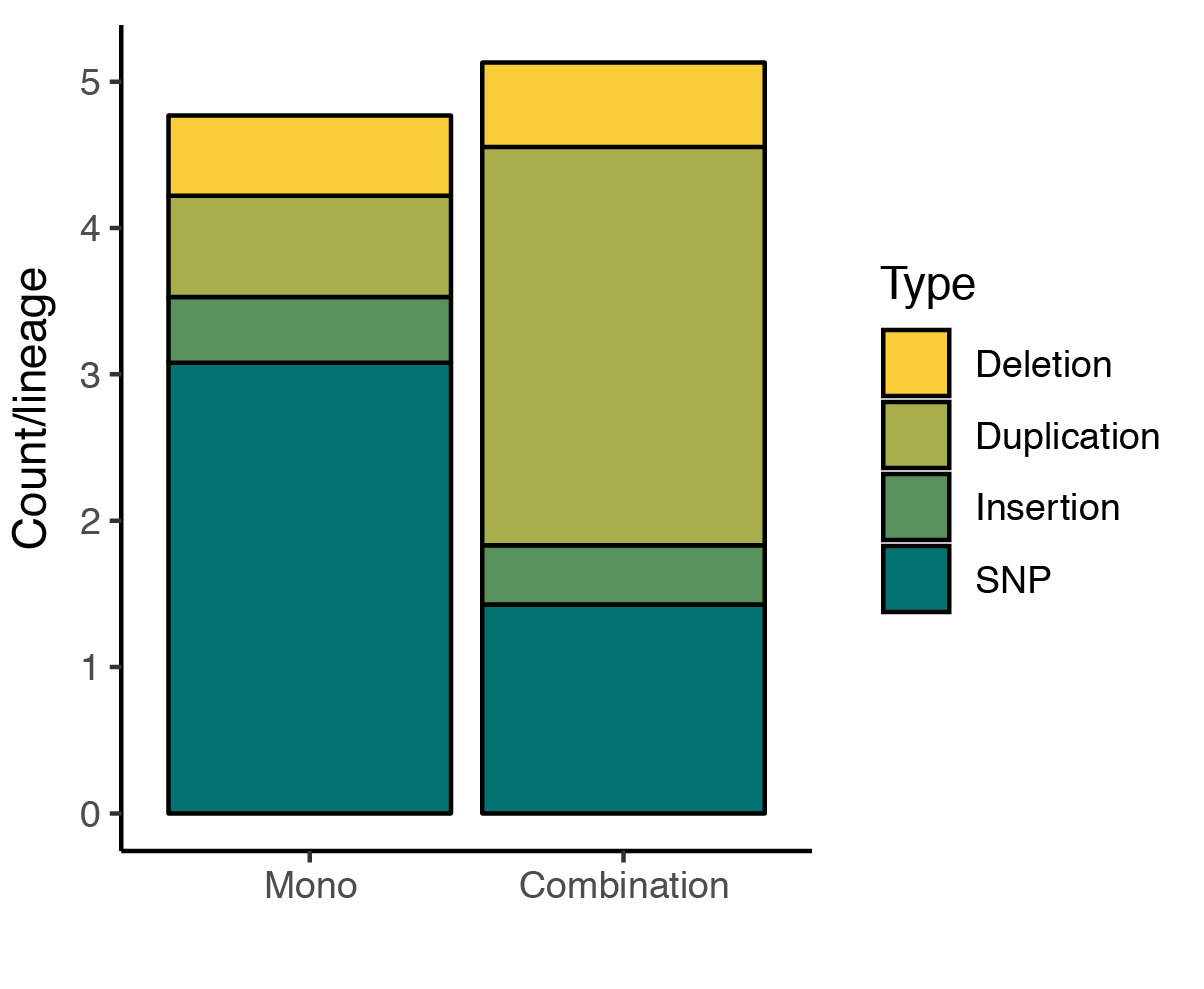
**

**Figure S4:** Count of different genetic adaptations normalized by the number of sequenced lineages in single drug evolved (Mono) and drug combination (Combination) exposed lineages.

**Fig. S5**

**
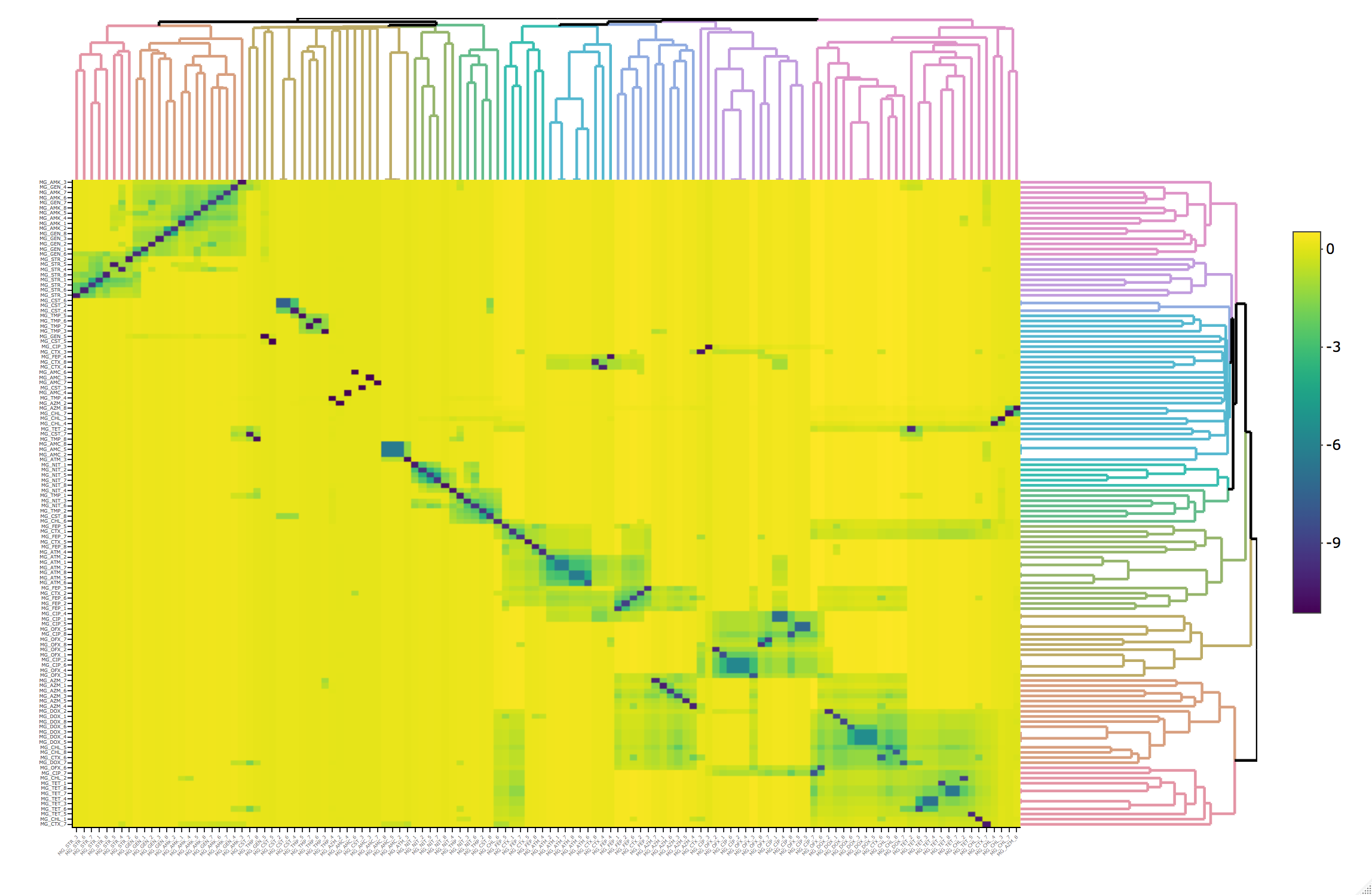
**

**Figure S5:** **Shared resistance mechanisms across different antibiotic classes**. Scaled (0-10) Jacquard’s Distance between the isolates evolved to single antibiotics is displayed from blue (identical) to yellow (very dissimilar). Hierarchical clustering groups isolates evolved to antibiotics with different mechanism of action together based on the genetic similarity, highlighting shared resistance mechanisms across different antibiotic classes. List of different drugs per cluster (top from left to right): Red: Streptomycin; Orange: Gentamicin, Amikacin; Beige: Colistin, Gentamicin, Trimethoprime, Azithromycin, Amoxicillin, Aztreonam; Olive: Nitrofurantoin; Green: Nitrofurantoin, Trimethoprime, Colistin, Chloramphenicol; Light turquoise: Cefepime, Cefotaxime, Aztreonam; Dark turquoise: Cefepime, Cefotaxime, Aztreonam; Blue: Cefepime, Cefotaxime, Azithromycin; Purple: Ciprofloxacin, Ofloxacin; Pink: Ciprofloxacin, Ofloxacin, Doxycyline, Tetracycline, Cefotaxime, Chloramphenicol, Azithromycin.

**Fig. S6**

**
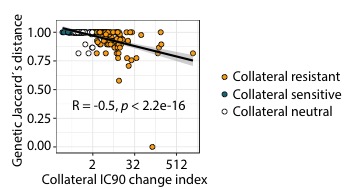
**

**Figure S6:** **Genetic similarity is significantly correlated with collateral resistance.** The genetic similarity between single drug evolved replicate lineages of drugs that constitute drug pairs was calculated and plotted against their Collateral IC_90_change index. The colors indicate collateral resistance (Collateral IC_90_ change index > 2, orange), collateral sensitivity (Collateral IC_90_ change index < 0.5, turquois), and collateral neutral (Collateral IC_90_ change index 0.5-2, white).

**Fig. S7**


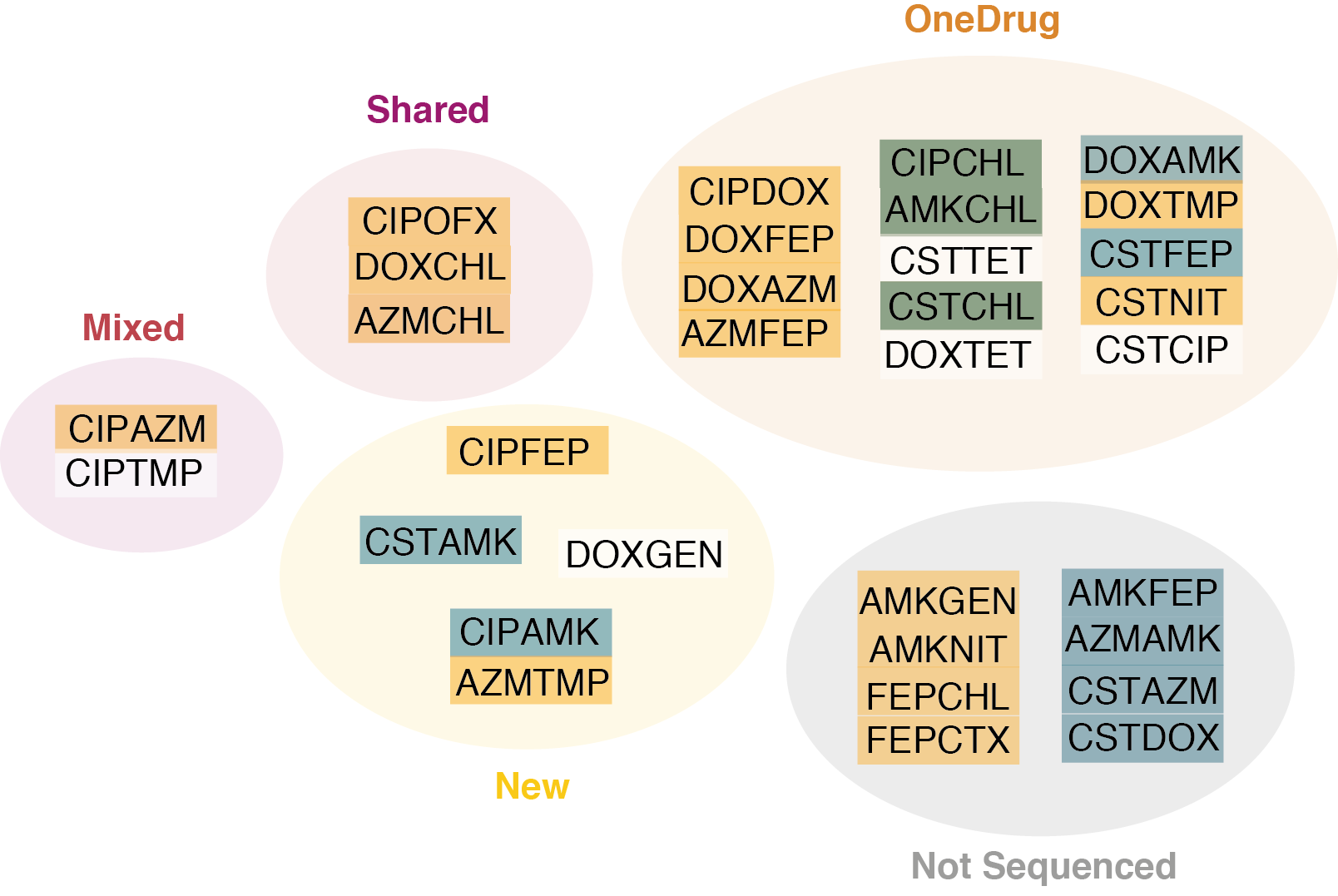


**Figure S7:** Distribution and features of drug pairs grouped based on genotypic response. The Mixed group contains drug pairs with neutral (white) or collateral resistance (orange) interactions. The Shared group contains only drug pairs with collateral resistance to each other. The OneDrug group is the largest group. The New group contains CIPFEP, which also selected for efflux mutations; therefore, grouping into the Shared group would be appropriate. The other drug pairs exhibited mainly collateral sensitivity (turquoise). Eight drug pairs were not sequenced due to failure in an initial resistance check. Half of these pairs are collateral resistant, while the other half are collateral sensitive.

**Fig. S8**

**Figure S8:** WT inhibition level of the individual drugs constituting a pair during the adaptive evolution experiment. In green the inhibition level of the first drug of the pair (Drug A) is displayed while the second drug is colored orange. The background of the drug pair names is colored according to the genetic group they belong to (Mixed, Shared, OneDrug, New and not sequenced).

**Fig. S9**

**Figure S9:** IC_90_ of the replicate lineages at four different time points during the adaptive evolution experiment. The different colors denote different parallel-evolved lineages. While resistance against Chloramphenicol is only selected after the drug exposure increases above the WT IC_90_ at day 7, resistance is already selected at subinhibitory concentrations under exposure to Ciprofloxain. This illustrates that the mutation selection window of different antibiotics can vary.

**Table S1**

All antibiotics and antibiotic pairs used for the adaptive evolution experiment.

|  | Antibiotic | Antibiotic Pair |  |
| --- | --- | --- | --- |
| 1 | Amoxicillin/Clavulanic acid | Amikacin + Cefepime |  |
| 2 | Piperacillin/Tazobactam | Amikacin + Chloramphenicol |  |
| 3 | Meropenem | Amikacin + Gentamicin |  |
| 4 | Ertapenem | Amikacin + Nitrofurantoin |  |
| 5 | Cefotaxime | Azithromycin + Amikacin |  |
| 6 | Cefepime | Azithromycin + Cefepime |  |
| 7 | Aztreonam | Azithromycin + Chloramphenicol |  |
| 8 | Ciprofloxacin | Azithromycin + Trimethoprim |  |
| 9 | Ofloxacin | Cefepime + Cefotaxime |  |
| 10 | Tetracycline | Cefepime + Chloramphenicol |  |
| 11 | Doxycycline | Ciprofloxacin + Amikacin |  |
| 12 | Amikacin | Ciprofloxacin + Azithromycin |  |
| 13 | Gentamicin | Ciprofloxacin + Cefepime |  |
| 14 | Streptomycin | Ciprofloxacin + Chloramphenicol |  |
| 15 | Azithromycin | Ciprofloxacin + Doxycycline |  |
| 16 | Erythromycin | Ciprofloxacin + Trimethoprim |  |
| 17 | Sulfamethoxazole | Ciprofloxacin + Ofloxacin |  |
| 18 | Trimethoprim | Colistin + Amikacin |  |
| 19 | Nitrofurantoin | Colistin + Azithromycin |  |
| 20 | Colistin | Colistin + Cefepime |  |
| 21 | Chloramphenicol | Colistin + Chloramphenicol |  |
| 22 | Fosfomycin | Colistin + Ciprofloxacin |  |
| 23 |  | Colistin + Doxycycline |  |
| 24 |  | Colistin + Nitrofurantoin |  |
| 25 |  | Colistin + Tetracycline |  |
| 26 |  | Doxycycline + Amikacin |  |
| 27 |  | Doxycycline + Azithromycin |  |
| 28 |  | Doxycycline + Cefepime |  |
| 29 |  | Doxycycline + Chloramphenicol |  |
| 30 |  | Doxycycline + Gentamicin |  |
| 31 |  | Doxycycline + Tetracycline |  |
| 32 |  | Doxycycline + Trimethoprim |  |
| 33 |  | Sulfamethoxazole + Trimethoprim | |

**Table S2**

This table contains a list of all antibiotics and their abbreviations, mechanisms of action and storage conditions.

**Table S3**

This table provides an overview of all antibiotic concentrations used during the ALE experiment.

**Table S4**

A list of all lineages and their last day of growth and day of revival can be found in this table.

**Table S5**

A list of all isolates with an evolvability index above 1 (except CIP-AZM) and potential genetic or technical explanations.

**Table S6**

A list of all isolates and whether or not they have been sequenced.

**Table S7**

A list of all INDELs, SNPs and gene duplications identified in the genome of each lineage after filtering out mutations that were also present in the media-adapted WT lineages.

**Table S8**

A table with all statistical information for the analysis of similarities (ANOSIM).

**Table S9**

A table with all phenotypic information regarding the drug-pair-evolved.
